# Emerging Biological Functions of IL-17A: A New Target in Chronic Obstructive Pulmonary Disease?

**DOI:** 10.3389/fphar.2021.695957

**Published:** 2021-07-02

**Authors:** Meiling Liu, Kang Wu, Jinduan Lin, Qingqiang Xie, Yuan Liu, Yin Huang, Jun Zeng, Zhaogang Yang, Yifan Wang, Shiyan Dong, Weiye Deng, Mingming Yang, Song Wu, Wen Jiang, Xuefeng Li

**Affiliations:** ^1^ The Sixth Affiliated Hospital of Guangzhou Medical University, Qingyuan People’s Hospital; State Key Laboratory of Respiratory Disease, Sino-French Hoffmann Institute, School of Basic Medical Sciences, Guangzhou Medical University, Guangzhou, China; ^2^ Shenzhen Luohu People’s Hospital, The Third Affiliated Hospital of Shenzhen University, Shenzhen, China; ^3^ South China Hospital, Shenzhen University, Shenzhen, China; ^4^ Department of Radiation Oncology, The University of Texas Southwestern Medical Center, Dallas, TX, United States; ^5^ Department of Radiation Oncology, The University of Texas MD Anderson Cancer Center, Houston, TX, United States

**Keywords:** IL-17A, COPD, inflammation, cytokine, chemokine, treatment

## Abstract

Chronic obstructive pulmonary disease (COPD) is a chronic inflammatory disease that causes high rates of disability and mortality worldwide because of severe progressive and irreversible symptoms. During the period of COPD initiation and progression, the immune system triggers the activation of various immune cells, including Regulatory T cells (Tregs), dendritic cells (DCs) and Th17 cells, and also the release of many different cytokines and chemokines, such as IL-17A and TGF-β. In recent years, studies have focused on the role of IL-17A in chronic inflammation process, which was found to play a highly critical role in facilitating COPD. Specially, IL-17A and its downstream regulators are potential therapeutic targets for COPD. We mainly focused on the possibility of IL-17A signaling pathways that involved in the progression of COPD; for instance, how IL-17A promotes airway remodeling in COPD? How IL-17A facilitates neutrophil inflammation in COPD? How IL-17A induces the expression of TSLP to promote the progression of COPD? Whether the mature DCs and Tregs participate in this process and how they cooperate with IL-17A to accelerate the development of COPD? And above associated studies could benefit clinical application of therapeutic targets of the disease. Moreover, four novel efficient therapies targeting IL-17A and other molecules for COPD are also concluded, such as Bufei Yishen formula (BYF), a Traditional Chinese Medicine (TCM), and curcumin, a natural polyphenol extracted from the root of *Curcuma longa*.

## Introduction

Chronic obstructive pulmonary disease (COPD), which is characterized by airflow obstruction and gas trapping of chronic bronchitis and emphysema, causes high morbidity and mortality. It can further develop into pulmonary heart diseases and common chronic diseases that result in severe respiratory failure (Adeloye et al., 2015). The common comorbidities of COPD include cardiovascular diseases, metabolic syndromes and lung cancer, leading to a worse health condition (Negewo et al., 2015). The World Health Organization (WHO) predicts that COPD will become the third leading cause of human death worldwide by 2030, pushing us to focus on the improvement of the treatments and prognosis.

The common clinical symptoms of COPD include chronic cough, sputum production, and dyspnea. The major risk factors are tobacco smoking, air pollution from indoors or outdoors, occupational dust, chemicals like smog, and infections caused by bacteria, viruses, or fungi. Besides environmental exposures, host factors such as abnormal lung development and genetic abnormalities are also important factors for individuals to develop COPD. COPD exacerbation is an important and complex event in COPD development and is a major determinant reflecting health status of COPD patients. According to the Global Initiative for Chronic Obstructive Lung Disease (2021), an COPD exacerbation is defined as an acute worsening of respiratory symptoms which requires additional therapy (Halpin et al., 2021). The common symptoms of COPD exacerbation include increased airway inflammation, increased mucus production and marked gas trapping, which leads to increased dyspnea, a key symptom of COPD exacerbation. Other symptoms include increased sputum purulence and volume, as well as increased cough and wheeze (Anthonisen et al., 1987). COPD exacerbation can be triggered by various factors, among which respiratory viral and bacterial infections are the most common factors. Meanwhile, environmental factors such as pollution and ambient temperature also initiate and/or amplify these events (Woodhead et al., 2005). The increase of bacteria burden in the sputum, eosinophil numbers together with neutrophils and other inflammatory cells are also the pathological features in a proportion of subjects with COPD exacerbation (Boixeda et al., 2015; Kim and Aaron, 2018). Studies conducted in smoking mice have shown that innate immunity plays a major role in the early stage of lung tissue changes, while acquired immune responses are important in the late stages of COPD (Rovina et al., 2013). Airway inflammation, which may induce parenchymal tissue destruction, disruption of defense mechanisms, and host normal repair, is a core feature in patients with COPD (de Nijs et al., 2011). Activating immune cells by stimulating inflammatory cell surface molecules is required to promote the development of lung inflammation in COPD patients (Wang et al., 2018a).

**FIGURE 1:**
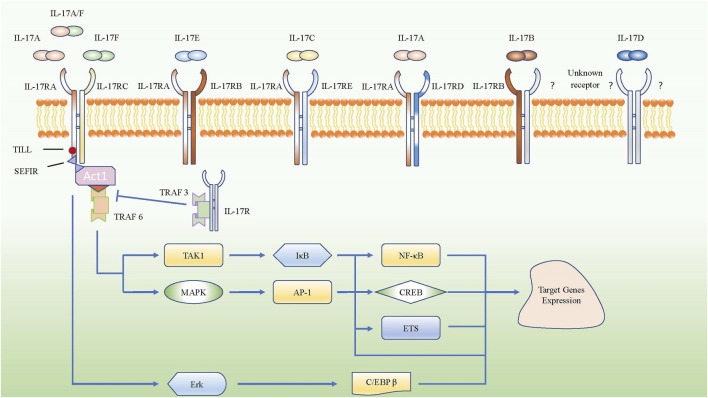
Schematic represent IL-17 family subgroup and their corresponding receptors from IL-17 receptor family. IL-17A, IL-17A/F and IL-17F share the IL-17RA/RC heterodimeric receptor. The intracellular domains of all IL-17R encode conserved SEFIR domains which interact with a corresponding SEFIR motif on the adaptor Act1, then they combine with TRAF6 to activate the pathway of NF-κB and MAPK. However, TRAF3 could inhibit SEFIR- Act1- TRAF6 pathway by competitive binding the IL-17RA directly. Only IL-17RA own the CBAD and TILL, which can promote the target gene expressions *via* C/EBP β pathway.

Interleukin-17 A (IL-17A), an essential member of the IL-17 family and known as an important proinflammatory factor in COPD, was cloned from activated cytotoxic T cells and originally named as CTLA8 (Rouvier et al., 1993). IL-17 can be produced by many different cells, such as Th17 cells, γδ T cells, innate lymphoid cells, lymphoid tissue inducer cells, and natural killer T cells (He et al., 2016). IL-17 participates in many important physiological responses (McGeachy et al., 2019). Naive T cells can differentiate into Th17 cells by up-regulating retinoic acid receptor-related orphan nuclear receptor γ (RORγt) mRNA by co-stimulating transforming growth factor-β (TGF-β) plus IL-6; IL-1β and IL-23 strengthen this process (Bettelli et al., 2006; McGeachy et al., 2009). Based on sequence homology, researchers later discovered that the IL-17 family includes six members: IL-17A, IL-17B, IL-17C, IL-17D, IL-17E (also called IL-25), and IL-17F. Till now, IL-17A is the most widely studied molecule in the IL-17 family and was found to have the closest relationship with IL-17F, among all family members. Besides, the receptor family of IL-17 consists of IL-17RA, IL-17RB, IL-17RC, IL-17RD, and IL-17RE. IL-17A shares the same IL-17RA/RC heterodimeric receptor complex with IL-17F and IL-17A/F heterodimers, while IL-17E binds to the heterodimeric receptor complex of IL-17RA and IL-17RB. IL-17C interacts with the heterodimeric receptor complex of IL-17RA and IL-17RE, and the combination of ligands and receptors contributes to passing the signal to the downstream pathways. However, the ligands for the heterodimeric receptor complex of IL-17RA and IL-17RD are not clear, though a study about psoriasis-like skin inflammation showed that IL-17RA/RD directly bound to IL-17A but not IL-17F or IL-17A/F heterodimer to mediate the proinflammatory gene expression downstream of IL-17A (Su et al., 2019). Besides, the receptor for IL-17D and the other component of IL-17RB heterodimeric receptor complex for IL-17B are still unknown (McGeachy et al., 2019). Actually, IL-17 receptor family share the SEFIR domain which is akin to Toll/Il-1 Receptor (TIR) domain, it can mediate SEFIR-SEFIR homotypic interactions by combing with Act1 (Novatchkova et al., 2003), then the combination of IL-17 receptor and Act1 recruits TNF receptor associated factor (TRAF) family members to initiate the pathways of nuclear factor kappa-B (NF-κB) and mitogen-activated protein kinases (MAPKs), resulting in the transcription and expression of target genes (Zhou et al., 2014; Li et al., 2016a; Herjan et al., 2018). Moreover, IL-17RA have the Toll/IL-1R-like loop (Till) and C/EBP-β activating domain (CBAD), which can activate extracellular signal-regulated kinase (Erk), leading to the phosphorylation of C/EBP-β and expression of target genes (Shen et al., 2009) (Figure 1).

Under normal condition, IL-17 protects the host against extracellular fungal and bacterial infections (Yang et al., 2018). However, IL-17A can also promote the progression of inflammatory processes, autoimmune diseases, chronic diseases like COPD and cancers, even worsen outcomes. Therefore, IL-17A and its family members are double-edged swords. On one hand, IL-17A can expand the role of the immune response in protecting the body against infections and promote tissue repair; on the other hand, the excessive secretion of IL-17A induces the expression of plenty inflammatory factors, which may result in conditions such as decreased flexibility of tissue and tissue fibrosis (Lei et al., 2019). Given the double effects of IL-17A, exploration the relationship and mechanism between IL-17A and various diseases is of essential importance.

The increased expression of IL-17A in the lung tissues of COPD patients was found to be negatively associated with the clinical evaluation indexes of lung function, such as forced expiratory volume in 1 s (FEV1%), forced vital capacity (FVC %) and FEV1/FVC (Zheng et al., 2018). Since FEV1%, FVC % and FEV1/FVC are negatively correlated with COPD severity (JalusicGluncic, 2011), the expression of IL-17A is positively correlated with the COPD severity. Although both IL-17A and IL-17F share the same receptor, their expression levels are different in COPD, while the affinity of IL-17F to IL-17RA/IL-17RC is far lower than that of IL-17A. Therefore, some researchers agree that IL-17A, rather than IL-17F, regulates the epithelial cell proliferation and apoptosis in central and distal airways to repair the damaged tissue in COPD patients (Eustace et al., 2011; Montalbano et al., 2015). Currently, it is generally believed that emphysema and severe airway obstruction are common clinical characteristics of COPD (Huang et al., 2018), and their evolvement and lymphoid neogenesis could be promoted by the up-regulation of IL-17A in COPD (Shan et al., 2012; Roos et al., 2015). Th17 cells, rather than IL-17A-secreting lung γδT cells, act as mediators of smoke-induced lung inflammation and emphysema. Unexpectedly, γδT cells can even hamper Th17 pathological responses in lung changes under certain conditions (Shan et al., 2012). Study has shown that lung inflammation in COPD is characterized by ascending neutrophils, alveolar macrophages, DCs, and T lymphocytes (predominantly TC1, TH1, and Th17 cells, while high level of eosinophils could also be detected in some patients with COPD (Barnes, 2016). Because IL-17A, a well-known pro-inflammatory factor in COPD patients, can promote the infiltration of inflammatory cells in the lung parenchyma and airways, the role of IL-17A in regulating the progression of COPD should be further explored.

## IL-17A Promotes COPD Progression

### IL-17A Promotes Airway Remodeling in COPD

**FIGURE 2:**
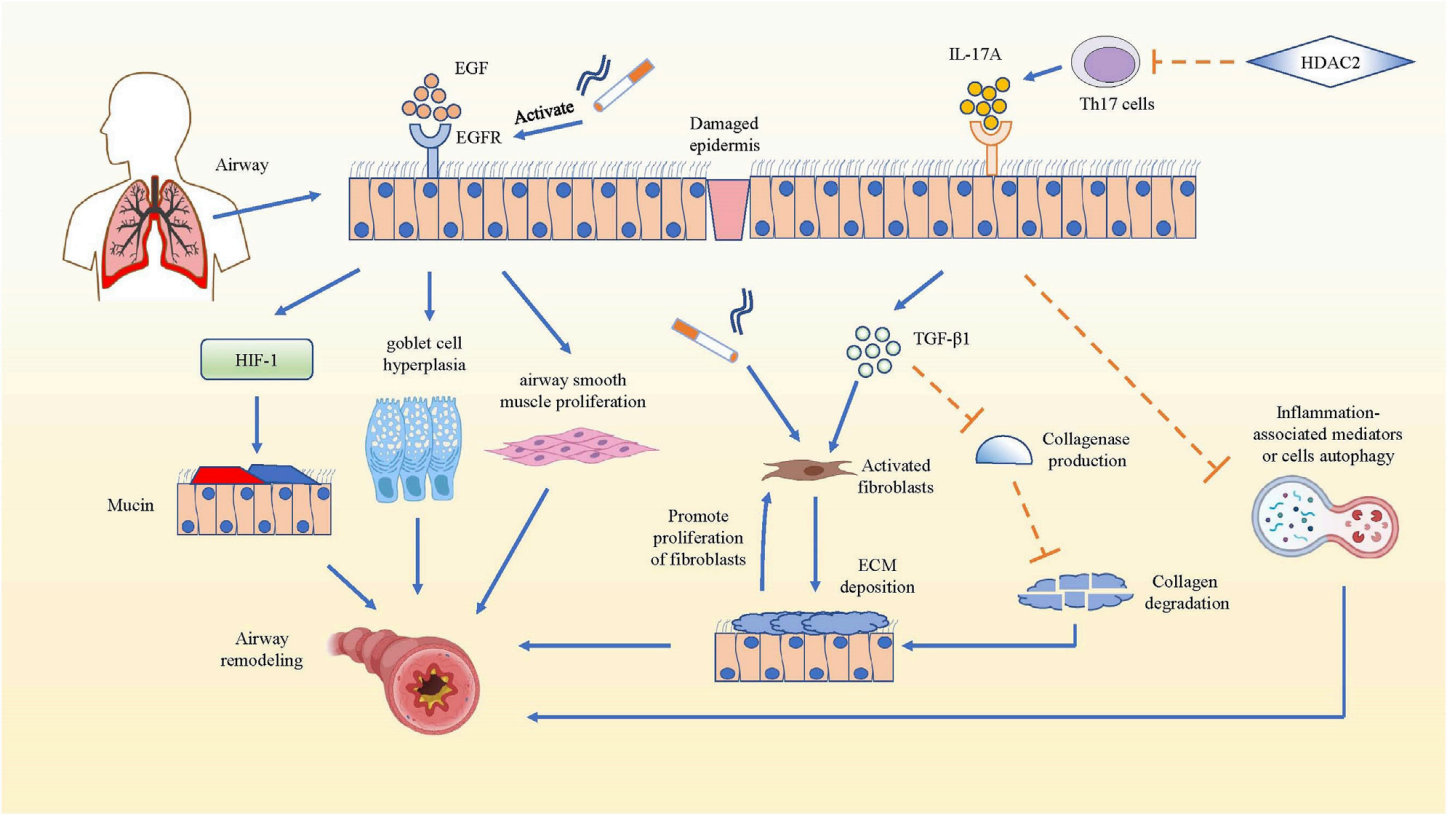
IL-17A promotes airway remodeling in COPD. The mechanism that IL-17A promotes airway remodeling by increasing collagen deposition and inhibiting inflammation-associated mediators or cells autophagy are summarized. In COPD airway remodeling, the activated EGFR facilitates the production of mucin5AC *via* hypoxia inducible factor-1 (HIF-1) pathway, promotes goblet cell hyperplasia and airway smooth muscle proliferation. While IL-17A produced by Th17 cells activates fibroblasts to secrete ECM and inhibit the collagen degradation in TGF-β1-dependent manner, and IL-17A also could hampers the autophagy of inflammation-associated mediators, which promotes development and progression of pulmonary fibrosis. Actually, the deposition of ECM also has positive effect on the proliferation of fibroblasts, which strengthens ECM deposition. However, HDAC2 reverses the above result by inhibiting the differentiation of Th17 cells.

Airway remodeling which could result in irreversible airflow obstruction is a common change in COPD lung tissue structure (Barnes et al., 2015). In airway remodeling, elevated level of epidermal growth factor (EGF) and increased expression of EGF receptors can be observed in the bronchial epithelial cells (Li and Luan, 2010). Cigarette smoke (CS), acting as an inducement for inflammation in COPD, can activate the EGF receptor through both damaged tissue and augmented epithelial permeability (Wang et al., 2014). The activated EGF receptor could promote the expression of airway mucin5AC and mucin5B, and facilitate goblet cell hyperplasia (Yu et al., 2012; Kato et al., 2020), while EGF could stimulate the proliferation of airway smooth muscle in the bronchi (Song et al., 2000). In fact, increased deposition of extracellular matrix (ECM) is an important structural change in airway remodeling, while fibroblasts are the main source of ECM and play a significant role in airway fibrosis formation, both of which play a key part in COPD airway remodeling (Hogg et al., 2004; Krimmer et al., 2012). In mouse pulmonary inflammation and fibrosis models, IL-17A not only directly regulated the synthesis and secretion of collagen in alveolar epithelial cells in a TGF-β1-dependent manner and facilitated the transition of epithelia to the mesenchyme, but also suppressed autophagy and promoted autophagy-associated cells death in inflammatory lung tissue (Li et al., 2015; Ye et al., 2015; Li et al., 2016b; Zhuang et al., 2016; Wang et al., 2018b). This led to more serious inflammation and possible airway remodeling, because the collagen and inflammation-associated mediators or cells could not be degraded or killed through autophagy (Mi et al., 1950; Xie et al., 2020). It has been reported that both IL-17A and Histone Deacetylase 2 (HDAC2) are related to the thickened wall and increased collagen deposition of the bronchi in COPD, but they have opposite effects. IL-17A can activate fibroblasts by inducing inflammatory cells to produce pro-fibrosis factor TGF-β1, which aggravates CS-induced airway remodeling, whereas HDACs could inhibit the differentiation of Th17 cells by reversing the high acetylation of core histone proteins. Therefore, the production of IL-17A from Th17 cells was reduced, and the airway remodeling in COPD was attenuated even inhibited (Lai et al., 2018). In all, IL-17A can promote airway remodeling by increasing TGF-β1 and inhibiting inflammation-associated mediator or cell autophagy in inflammatory lung tissue (Figure 2).

### IL-17A Facilitates Neutrophil Inflammation in COPD

COPD is a chronic inflammatory airway disease dominated by neutrophil infiltration. In COPD, increased neutrophil mobilization and elevated IL-17A were observed. Although CS is regarded as one of the crucial causes for triggering COPD, IL-17A is not significantly reduced after smoking cessation (Hansen et al., 2014). IL-17A, p53, and plasminogen activator inhibitor (PAI)-1 were higher in smokers with COPD than in healthy smokers (HSs) and healthy control subjects (HCs) (Gouda et al., 2018a). P53, a tumor suppressor gene that controls the initiation of the cell cycle, could initiate cell apoptosis if damage to cells is beyond repair. As COPD pulmonary fibrosis injury is associated with damaged alveolar epithelia, research has mostly focused on damaged alveolar epithelia and found higher expression of p53 and PAI-1, as well as the activation of caspase-3, which participates in promoting apoptosis of alveolar epithelial cells (Bhandary et al., 2013). In the experiment of bleomycin-induced alveolar basal epithelial cells to simulate the inflammation *in vitro*, the up-regulation of IL-17A promoted migration of alveolar basal epithelial cells and increased p53 and PAI-1 expression (Gouda et al., 2018b). p53 can induce obvious epithelial cell apoptosis and lung injury, while PAI-1 is a downstream mediator of p53-induced pulmonary inflammation. After serine phosphorylation in the p53 protein, p53 binds to PAI-1 to promote PAI-1 expression, leading to the increase of CXCL1, CXCL2 and CXCR2 in COPD patients (Tiwari et al., 2016), which could induce the influx of neutrophils to infection site. Higher PAI-1 levels can not only promote airway and alveolar epithelial cells apoptosis, but also cause fibrinolysis defects and alveolar fibrin deposition in patients with COPD (Gouda et al., 2018a). Meanwhile, the increased expression of PAI-1 could suppress neutrophil apoptosis and apoptotic neutrophil phagocytosis (Zmijewski et al., 2011), leading to neutrophil accumulation and inflammation in damaged lungs of patients with COPD.

**FIGURE 3:**
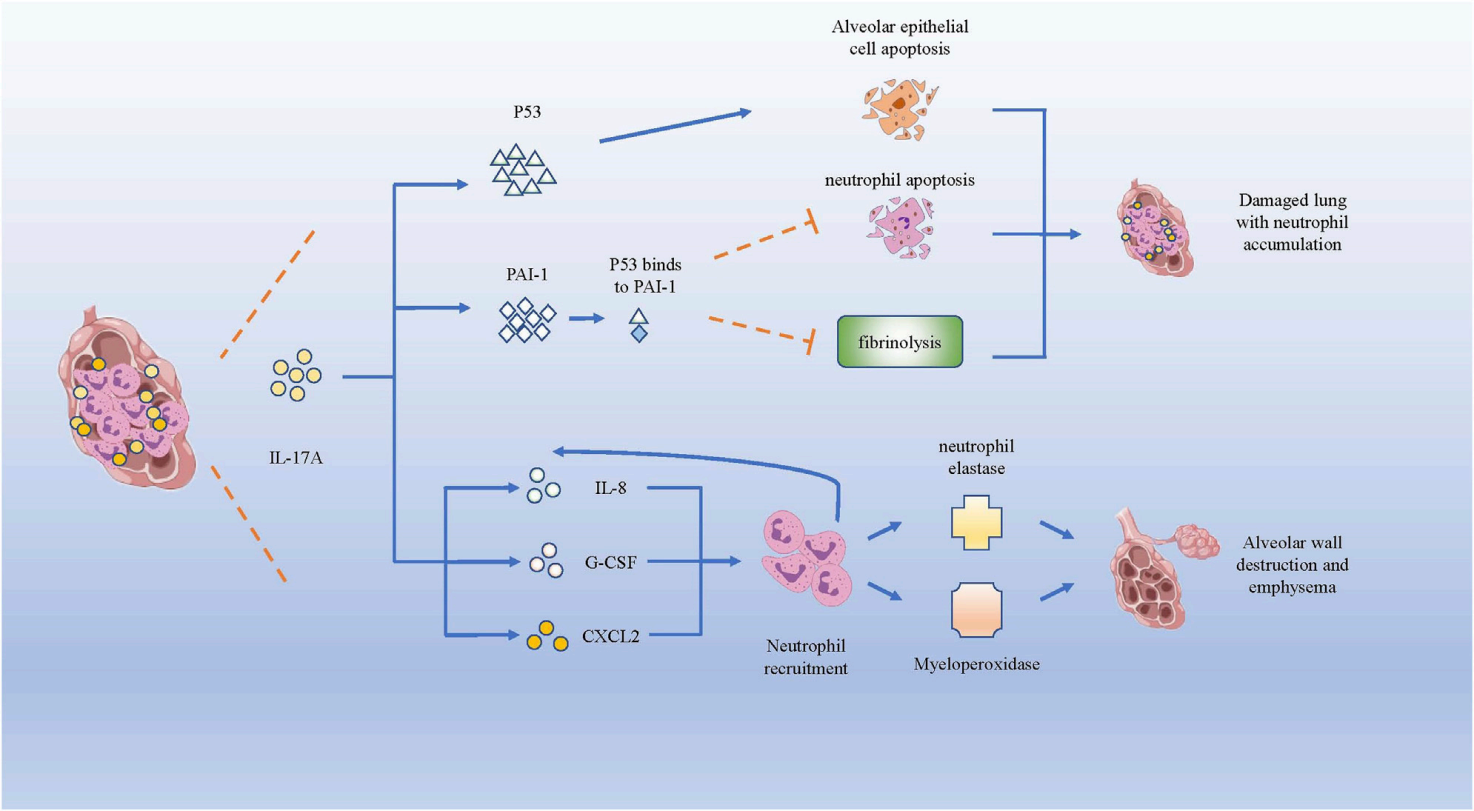
IL-17A promotes neutrophils infiltration and lung destruction by increasing p53 and PAI-1. Both p53 and PAI-1 can induce the apoptosis of alveolar epithelial cells, while PAI-1 inhibits the neutrophil apoptosis and fibrinolysis in lung tissue. Besides, IL-17A induces the expression of IL-8, G-CSF and CXCL2, which recruits neutrophils producing neutrophil elastase and myeloperoxidase, giving rise to the destruction of alveolar wall and emphysema.

In brief, IL-17A could facilitate neutrophil inflammation in COPD by facilitating the expression of p53 and PAI-1 to increase CXCL1, CXCL2 and CXCR2 to induce the influx of neutrophils. Besides, IL-17A can recruit neutrophils to the inflammation nidus by inducing the expression of neutrophil chemokines, such as IL-8, G-CSF and CXCL2 (Hansen et al., 2014). The increase of neutrophils could secrete more neutrophil elastase (NE) and myeloperoxidase, which can degrade collagen, destroy alveolar wall and cause emphysema (Cohen et al., 1982) (Figure 3).

### IL-17A Induces TSLP Expression by Activating IKK-α in COPD

Thymic stromal lymphopoiesis (TSLP), a cytokine homologous to IL-7, is expressed in epithelial cells and is involved in the pathogenesis of chronic diseases by combining with its receptor (El-Ghareeb et al., 2019). TSLP can directly promote the differentiation as well as the response of Th2 cells in the lung to promote COPD through an independent pathway without draining lymph nodes (Lai et al., 2020). TSLP-activated DCs primed naïve T(H) cells to produce cytokines such as IL-4, IL-5, IL-13 and TNF-α to facilitate the development of Th2 cell-mediated airway inflammation (Soumelis et al., 2002). Research on TSLP production in COPD patients has shown that serum concentrations of TSLP and IL-17A were higher in patients with COPD than that in HCs. After stimulating a human epithelial cell line (16HBE) with induced sputum supernatants (ISS) from HCs, HSs, or COPD patients, significantly higher levels of TSLP were induced by elevated IL-17A from COPD patients, which could be inhibited by anti-IL-17A Ab(antibody), indicating a direct and specific association between IL-17A and TSLP in the airways. IKKα silencing reduced TSLP synthesis in 16HBE stimulated with IL-17A or with ISs from COPD patients more effectively than in stimulated unsilenced cells (Anzalone et al., 2018). Therefore, IL-17A, TSLP, and IKKα might closely interact. Anzalone et al. reported that IL-17A induced the acetylation of histone H3 (Ac-His H3) (k9) and inflammation by activating IKKα in human lung epithelial cells (Anzalone et al., 2016). In conclusion, the aforementioned studies suggested that IL-17A initiated IKK-α signaling to induce the TSLP production to regulate airway inflammation in COPD.

The specific induction process mentioned earlier may involve CREP-binding protein (CBP), NF-κB, and IκB kinase, so their roles and functions need to be elucidated first. IKK-α is a catalytic subunit of the IκB kinase (also named IKK complex, which consists of IKK-α, IKK-β, and IKK-γ), and NF-κB is a key nuclear transcription factor related to activating immune cells, and also to triggering T and B cells, apoptosis, and stress response (Liu et al., 2012). At rest, NF-κB binds to the IκB kinase, presenting an inactive status. Upon external stimulation, the IKK complex becomes active and NF-κB is released from the complex. Then, the activated NF-κB translocates from the cytoplasm to the nucleus, and acts as a key nuclear transcription factor to regulate the expression of various genes, including those of cytokines and chemokines associated with airway inflammation (Mulero et al., 2019). Interestingly, IKK-β, but not IKK-α, plays a critical role in cytokine-induced IκB activation of the classical pathway to regulate the transcription of various cytokines and chemokines. Nevertheless, IKK-α-deficient mice are defective in their ability to induce NF-κB-dependent transcription. Therefore, a direct key relationship between IKK-α and NF-κB is still unclear. Studies later found that IKK-α can also directly translocates to the nucleus to rapidly regulate the expression of NF-κB response gene by catalyzing phosphorylation of histone H3 to activate NF-κB-directed gene expression (Yamamoto et al., 2003).

**FIGURE 4:**
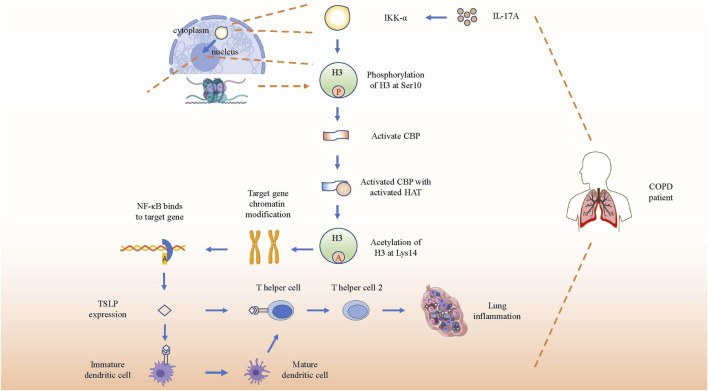
IL-17A induces gene expression by activating IKK-α. Schematic show the pathway of inflammation-associated gene expression directly regulated by activated IKK-α through direct translocation into nucleus after receiving the stimulation of increased IL-17A. After phosphorylation and acetylation of H3, the target genes’ chromatin will be recombined and bind to NF-κB to promote TSLP expression. Then TSLP will combine with its receptor on immature dendritic cells and T helper cells to promote the differentiation and immune response of Th2 in COPD lung tissue.

Hence, inducing TSLP and other pro-inflammatory mediators by IL-17A-associated IKK-α signaling in patients with COPD can be described as follows. First, elevated IL-17A in COPD patients activates IKK-α. Second, IKK-α catalyzes phosphorylation of Ser10 in histone H3 to activate CBP. Third, the activated CBP that owns activated histone acetyltransferase (HAT) activity acetylates H3 at Lys14, contributing to chromatin modification such as dissociating DNA and histone octamer, so that a variety of transcription factors such as NF-κB can specifically combine with DNA binding sites and activate gene transcription, thereby promoting the expression of pro-inflammatory molecules such as TSLP in patients with COPD (Chung et al., 2010; Anzalone et al., 2018) (Figure 4). In brief, IL-17A promotes the expression of TSLP and other pro-inflammatory mediators by activating IKK-α to drive the development of COPD.

### DCs Induces the Differentiation of Th17 Cells to Produce More IL-17A to Promote COPD Progression

DCs are functional specialized antigen-presenting cells. Different biomarkers indicate a different maturity DC status. For example, CD1a and CCR6 are used for immature DCs, and CD40, CD80, and CD83 are used for mature DCs.

Zheng et al. confirmed that levels of CD80, Treg, FOXP3, and mature DCs were significantly lower, and levels of CCR6, Th17 cells, IL-17A, and immature DCs were higher in COPD^+^ patients than in CS^+^ COPD^−^ patients and HCs. The acute exacerbation of the COPD (AECOPD) group showed the highest effects on decreasing mature DCs and increasing immature DCs (Zheng et al., 2018). Several studies have indicated that DCs induced CD4^+^ T cells to differentiate toward a Th17 cell type by up-regulating RORγt mRNA *via* the CD40/CD40L pathway and cooperating with IL-6 and IL-23 that were produced by DCs (Perona-Wright et al., 2009; Iezzi et al., 2009). Higher levels of Th17 cells not only produce more IL-17A to enhance inflammation, but also induce the differentiation and activation of DCs, which, in turn, further induce the differentiation of Th17 cell and strengthen the inflammation response in COPD (Vargas-Rojas et al., 2011; Wang et al., 2019). In brief, the increase of IL-17A produced by DCs-induced Th17 cells could promote the progression of COPD, which suggests that the subgroup of DCs plays an important role in promoting COPD development.

### Imbalance of Th17/Treg in COPD Progression

Tregs are a subgroup of T cells with strong immunosuppressive functions and Th17 are the T helper cells that could secret IL-17A.

Wang et al. reported that COPD patients had higher levels of circulating Th17 cells, serum IL-1β, IL-6, IL-17A, IL-21, IL-22, IL-23, and TGF-β, but lower levels of Tregs and IL-10, which accounted for the dramatically higher ratio of Th17 to Tregs than that in HCs. Interestingly, they also observed that Tregs counts were remarkable higher in HSs, but lower in patients with COPDs, than in HCs. This phenomenon suggested the participation of Tregs in the normal immune system to sustain physiological homeostasis by functioning in immune-suppression. Simultaneously, the same tendency of fork head/winged helix transcription factor (FOXP3) mRNA and IL-10 levels were also observed in these groups (Wang et al., 2015a). Similarly, Ito et al. used CS-induced COPD mice to prove the increased expression of pro-inflammatory molecules such as TNF-α, IL-6, and IL-17 along with decreased anti-inflammatory molecule and cell levels such as IL-10 and FOXP3. These changes were linked to higher alveolar enlargement and impaired lung function in CS-induced COPD mice than healthy control mice (Ito et al., 2019).

Despite increased IL-2 and TGF-β (also considered to be a type of anti-inflammatory chemokine that promotes Tregs expression) in patients with COPD and elevated Tregs frequencies in AECOPD (Jin et al., 2014), higher levels of pro-inflammatory molecule such as IL-1β, IL-6, and IL-23 were predominant in patients with COPD. Because IL-1β, IL-6, and IL-23 can strongly promote the expression of Th17 cells but inhibit FOXP3 and Tregs (Morishima et al., 2009), we can easily account for the increased Th17 and decreased Tregs in COPD. Besides, elevated levels of IL-21 also down-regulated Tregs differentiation (Nguyen et al., 2012) and regulated Th17 development in a critical autocrine way (Nurieva et al., 2007). Although IL-22, an essential member of the IL-10 family, promotes tissue repair and host defense by binding to its heterodimeric transmembrane receptor complex composed of IL-22R1 and IL-10R2, it can also cooperate with co-expressed IL-17 to induce secretion of neutrophil chemotactic factors such as GM-CSF and CXCL8, and cytokines such as IL-6 to promote inflammation (Rutz et al., 2013).

Overall, elevated levels of pro-inflammatory molecules, such as IL-1β, IL-6, and IL-23, induced the development of Th17 cells, which, in turn, secreted pro-inflammatory chemokines and cytokines like IL-17A. IL-17A cooperated with other molecules like IL-22 to recruit GM-CSF, CXCL8 and IL-6 to promote inflammation development. In contrast, the suppressive development of Tregs and relative anti-inflammatory molecules certainly like IL-10 damaged their strong immunosuppressive function, thus both changes further promoted inflammation as well as COPD progression. Taking advantage of the imbalanced ratio of Th17 to Tregs, we can learn about the progression of COPD.

## Relative Important and Common Molecules Promoting COPD Development

### IL-17A Promotes the Expression of IL-8 in COPD

IL-8, also known as chemokine CXCL8, is secreted by macrophages, epithelial cells, among other cells. It can bind to IL-8RA (also named CXCR1) and IL-8RB (also named CXCR2), which are distributed on the surface of neutrophils, and the combination recruits and activates neutrophils to the damaged site to generate an inflammatory immune response (Lin et al., 2004). IL-8 has been proved to induce the recruitment and activation of neutrophils in the bronchial epithelium of COPD, and plays an important role in persistent airway inflammation of COPD and also reduces steroid sensitivity (Reynolds et al., 2018).

Anzalone et al. found higher levels of both macrophages and neutrophils in HSs, while only higher levels of neutrophils were observed in patients with COPD. The concentrations of IL-17A and IL-8 were dramatically elevated in ISS from patients with COPD. They detected the relative molecule expression in 16HBE after stimulating with ISS from HCs, HSs, and COPD patients, and the control group was untreated 16HBE. The expression of IL-8 mRNA and IL-8 protein were higher in 16HBE stimulated with ISS from HSs and COPD patients than that of 16HBE stimulated with the ISS from HCs or untreated 16HBE. Also, 16HBE stimulated with IL-17A yielded the same results, including increased IL-8, Ac-His H3 (k9), and IKKα, although 16HBE pretreated with anti-IL-17A or tiotropium could reverse this phenomenon (Anzalone et al., 2016).

According to above results, higher IL-17A level have promoted the expression of IL-8 in patients with COPD. A possible mechanism is that IL-17A facilitates the expression of inducible nitric oxide synthase (iNOS), which promotes the production of nitric oxide (NO) (Su et al., 2016). Furthermore, NO promotes the transcription of IL-8 mRNA by NF-κB and AP-1 to express higher IL-8 levels (Seo et al., 2009). These results indicated the positive link between IL-17A and IL-8 in patients with COPD, leading to neutrophil infiltration and airway inflammation.

### IL-1β Cooperate With Other Cytokines to Induce IL-17A Expression in COPD

IL-1β is mainly produced by monocytes and macrophages and can work with the IL-1 receptor (IL-1R), distributed on the surface of most nucleated cells, to promote monocytes to directionally migrate toward inflammatory lesions. Therefore, it is considered to be a potent pro-inflammatory cytokine that is crucial for host-defense responses to infection and injury (Dinarello, 1996). IL-1β can also promote the development of Th17 cells that produce IL-17A by cooperating with TGF-β, IL-6, and IL-23, contributing to the production of IL-17A in COPD progression when infections taking place (Revu et al., 2018).

## Prevention and Present Treatment

In order to prevent and improve the symptoms of COPD, individuals are recommended to stop smoking (van Eerd et al., 2016), perform pharmacological therapy, make use of inhaler technique, undergo pulmonary rehabilitation or/and inject influenza vaccination (Poole et al., 2006). For stable COPD, patients are long to alleviate the symptoms, reduce the frequency and severity of exacerbations, and improve exercise tolerance.

The American College of Physicians (ACP), American College of Chest Physicians (ACCP), American Thoracic Society (ATS), and European Respiratory Society (ERS) aim at managing patients with COPD, according to the following guidelines: *1*) Patients with respiratory symptoms and 60–80% FEV1 can use bronchodilators *via* inhalation. *2*) Patients with respiratory symptoms and FEV1<60% are recommended to use bronchodilators *via* inhalation. *3*) Patients with symptoms and FEV1<60% are recommended to use either long-acting anticholinergics or long-acting β-agonists, both *via* inhalation. *4*) Patients with symptoms and FEV1<60% could use combined therapies *via* inhalation (long-acting anticholinergics, long-acting β-agonists, or corticosteroids) *5*) Patients with symptoms and FEV1<50% are strongly recommended to receive pulmonary rehabilitation, while patients with symptoms or exercise-limitation and FEV1 >50% could use pulmonary rehabilitation. *6*) Patients with severe resting hypoxemia (Pao2 ≤ 55 mm Hg or Spo2 ≤ 88%) are strongly encouraged to receive continuous oxygen therapy (Qaseem et al., 2011).

Tiotropium, a kind of long-acting muscarinic antagonist (LAMA), can inhibit the activation of airway secretory cells and smooth muscle cells, which, in turn, can reduce mucus secretion in the lungs of patients with COPD to improve their function (Coulson and Fryer, 2003). Bronchodilation with LAMAs, such as tiotropium, and long-acting ß2-agonists (LABAs), such as olodaterol, is a highly effective long-term maintenance treatment for COPD. Anzalone et al. showed that pretreating 16HBE cells with tiotropium significantly reduced the expression of IL-8 and Ac-His H3 (k9), and decreased the translocation of nuclear IKKα protein after stimulation with ISS in patients with COPD, which indicated the therapeutic effectiveness of tiotropium in COPD (Anzalone et al., 2016).

Although the therapeutic methods aforementioned improve airflow limitation and suppress the inflammatory responses of COPD to some extent, side effects are still observed. For instance, inhaled corticosteroids may cause dysphonia and moderate to severe bruising, while long-acting inhaled β-agonists can lead to increased cardiovascular events; tiotropium may cause dry mouth in patients (Yohannes et al., 2011; Gershon et al., 2013). These results indicate the need for therapeutic methods with minimum adverse effects in patients with COPD.

### Novel and Promising Drug Treatments

As it is urgent for patients with COPD to get some effective and safe treatments, we introduce several novel and promising drug treatments as follows. Studies have shown the effectiveness of the Bufei Yishen formula (BYF), a Traditional Chinese Medicine (TCM) that consists of 12 medicinal herbs, as a potential therapeutic for COPD (Li et al., 2012). BYF can effectively alleviate COPD symptoms by declining exacerbation frequency, delaying acute exacerbation, and improving pulmonary function in patients. Later, Zhao et al. proved that BYF with oral administration could decrease the levels of pro-inflammatory cytokines such as IL-1β, IL-6, TNF-α, and IL-17A secreted by Th17 cells, but increase anti-inflammatory cytokines IL-10 secreted by Tregs in the BAL fluid. BYF treatment dramatically decreased CD4^+^RORγt^+^ T (Th17) cells and increased CD4^+^CD25^+^FOXP3^+^ T (Treg) cells, so that the balance of Th17/Tregs could be restored to slow COPD progression. Moreover, BYF further suppressed phosphorylation of signal transducer and activator of transcription-3 (STAT3) and promoted phosphorylation of STAT5 (Zhao et al., 2018). Similarly, Dong et al. proved that BYF inhibited the lipopolysaccharide - or CS extract -induced expressions of TNF-α, IL-8 in H292 cells, and suppressed the activation of transcription factors like NF-κB and STAT3 to inhibit their correlative pathways (Dong et al., 2020). STAT3 is known to be a critical transcription factor that induces RORγt gene expression to promote Th17 differentiation (Lee et al., 2017). In contrast, STAT5 is regarded as an important transcription factor that induces the development and maintenance of Tregs by binding to the FOXP3 gene (Ma et al., 2018), but inhibits the expression of Th17 cells (Zheng et al., 2015). In brief, BYF could improve COPD by restoring the Th17/Treg balance as well as increasing related anti-inflammatory cytokines, while decreasing pro-inflammatory cytokines. In fact, BYF can treat patients with COPD *via* their multiple components. According to the network pharmacology analysis and molecular docking validation, a study showed that 48 components in BYF might act on 65 targets to exert the therapeutic effect, and the therapeutic process involved IL-17, Toll-like receptor (TLR) and TNF pathways (Wu et al., 2020). Therefore, more attention should be drawn to BYF for therapeutic use in COPD.

**FIGURE 5:**
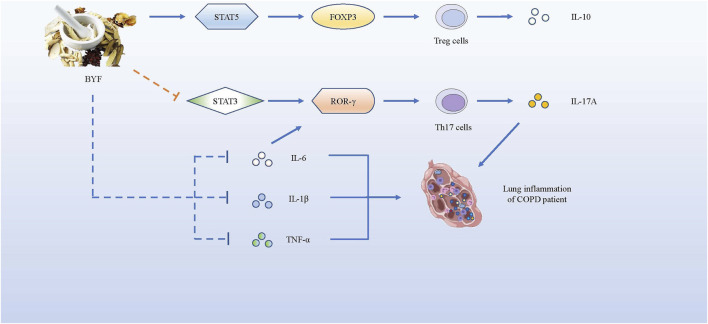
BYF alleviates COPD symptoms by promoting the expression of pro-inflammatory factors and suppressing the production of anti-inflammatory cytokines. BYF induces T naïve cells towards Tregs, producing IL-10, by up-regulating the FOXP3 expression *via* STAT5 pathway, while it inhibits the differentiation of Th17 cells, producing IL-17A, by down-regulating the RORγt gene *via* STAT3 pathway. Meanwhile, BYF also decrease the levels of pro-inflammatory cytokines such as IL-1β, IL-6 and TNF-α, which could alleviate the inflammation development of COPD.

Curcumin, a natural polyphenol extracted from the root of *Curcuma longa* (turmeric plant), is a safe and bioactive phytochemical component with many molecular targets and high pharmacological activity. Curcumin plays a key role in decreasing serum lipids, suppressing inflammation, promoting antitumor activities, and improving the development of COPD (Khorasani et al., 2019). A study showed that administrations of bleomycin and IL-17A to the alveolar epithelial cells could result in significant down-regulation of Akt expression, a proliferation biomarker for cell, while this phenomenon could be reversed by treatment with Curcumin (Gouda et al., 2018c). Another study indicated Curcumin alleviated IL-17A-mediated p53-PAI-1 expression in bleomycin-induced alveolar basal epithelial cells (Gouda et al., 2018b). Both of these two studies showed pulmonary inflammation mediated by bleomycin and IL-17A was intervened effectively by curcumin. Chen et al. further proved that Curcumin decreased the inflammation of acute lung injury not only by promoting the expression of IL-35, increasing STAT5 proportion, but also by decreasing IL-17A in lung tissue (Chen et al., 2020). Curcumin can significantly decrease the number of neutrophils and macrophages in the BAL fluid, and reduce the apoptosis index of alveolar epithelial cells to attenuate alveolar epithelial damage in COPD, and may be associated with down-regulating the expression of p66Shc (Zhang et al., 2016). Curcumin can also suppress chemokine expression by restoring HDAC2 expression and function on histone modification to attenuate COPD development (Gan et al., 2016). Also, curcumin effectively reduced CS-induced inflammation responses and improved pulmonary function, presumably by up-regulating peroxisome proliferators-activated receptor γ (PPARγ) and inhibiting NF-κB activation (Li et al., 2019). Therefore, curcumin is a safe and natural drug with promising therapeutic value for improving the life quality of patients with COPD (Figure 5).

MLN4924, a potent and selective inhibitor of NEDD8-activating enzyme (NAE), mediates cell cycle by deregulating the S-phase of DNA synthesis (Soucy et al., 2009). MLN4924 could significantly inhibit the expression of proinflammatory cytokines and chemokines such as IL-1β, IL-6, and CXCL-1 in IL-17A-inducing pulmonary inflammation. Mechanistically, MLN4924 inhibited TRAF6 ubiquitination by interfering the interaction between ACT1 of IL-17A and TRAFs, leading to significant inhibition of MAPK and NF-κB pathways (Hao et al., 2019). The other study also showed that MLN4924 acted against bleomycin-induced pulmonary fibrosis mainly at the early inflammatory stage, during which it inhibited MAPK and NF-κB pathways to decrease the expression of proinflammatory cytokines and chemokines. And the study indicated the potential therapeutic role of MLN4924 against other inflammation-associated diseases like COPD (Deng et al., 2017). However, although MLN4924 might be a novel and promising drug treatment for COPD, there are very few correlative studies at present, which needs our more attentions to study them.

Simvastatin, belonging to Statins, certainly has anti-inflammatory, anti-oxidant function *in vitro* and *in vivo*. Maneechotesuwan et al. showed that Simvastatin reversed the IL-17A/IL-10 imbalance in the airways of COPD. Compared with placebo treatment, IL-17A, IL-22, IL-6, and CXCL8 concentrations in sputum significantly reduced at 4 wk, whereas IL-10 were markedly increased during Simvastatin treatment (Maneechotesuwan et al., 2015). Another study proved that in rat COPD model, Simvastatin decreased the levels of IL-8, IL-17 and TNF-α in bronchoalveolar lavage fluid (BALF) and inhibit the expression of NF-κB and MUC5AC in airway and lung tissue. Simvastatin plays preventive and therapeutic roles by reducing airway inflammation and airway mucus hypersecretion (Wang et al., 2015b). Murphy et al. showed that IL-17 upregulated IL-8, IL-6, G-CSF and GM-CSF, whereas TGF-β increased IL-6 and GM-CSF. Simvastatin attenuated effects of both IL-17 and TGF-β. Their study has demonstrated the ability of Simvastatin to alleviate neutrophilic airway inflammation and remodeling by inhibiting the upregulation of various pro-inflammatory cytokines induced by IL-17A and TGF-β (Murphy et al., 2008). There are less associated studies about Simvastatin, targeting IL-17A, for COPD at present, which needs to be studied further.

Recently, Christenson et al. discovered a new COPD subgroup characterized by a markedly enhanced IL-17 signature that decreased in response to corticosteroids. This subgroup was irrelevant to airway eosinophilic or type 2 inflammation. Hence, more attention should be focused on exploring the relative specific mechanism of this COPD subgroup, so that we can improve personalized therapy to improve the life quality of patients with COPD (Christenson et al., 2019).

## Conclusion

COPD is a chronic inflammatory disease that leads to high rates of disability and mortality worldwide. Increased expression of IL-17A promotes the progression of COPD, but some specific underlying mechanisms which promote the development of this disease is still unknown. Hence, we looked into the relationship between IL-17A and immune cells, chemokines, cytokines, and transcription factors to explore and discuss several possible mechanisms of IL-17A in promoting COPD progression. We discussed some specific mechanisms of IL-17A in facilitating the progression of COPD, and introduced some promising effective therapies to improve the symptom of COPD. Further, in order to encourage more studies on the novel therapeutic targets for COPD, we provided some useful novel viewpoint for clinical applications of COPD therapy.

Based on all kinds of inducements for COPD, IL-17A can exert important function to promote the inflammation in patients with COPD. Firstly, IL-17A can promote airway remodeling by increasing TGF-β1 expression and inhibiting inflammation-associated mediators or cells autophagy in inflammatory lung tissue, while HDACs can inhibit the differentiation of Th17 cells by reversing the high acetylation of core histone proteins to reduce IL-17A from TH17 cells to alleviate airway remodeling. Secondly, IL-17A can facilitate neutrophil inflammation in COPD by facilitating the expression of p53 and PAI-1 to increase CXCL1, CXCL2 and CXCR2 to induce the influx of neutrophils towards nidus. Thirdly, IL-17A promotes the expression of TSLP and other pro-inflammatory mediators by activating IKK-α to drive the development of COPD. Fourthly, DCs induces CD4^+^ T cells to differentiate toward Th17 cells to produce more IL-17A to promote inflammation, which in turn further induces the differentiation and activation of DCs, leading to enhanced inflammation response in COPD. Last but not least, elevated levels of pro-inflammatory molecules such as IL-1β, IL-6, and IL-23, induce the development of Th17 cells, which, in turn, secrete pro-inflammatory chemokines and cytokines like IL-17A. IL-17A cooperated with other molecules like IL-22 to recruit GM-CSF, CXCL8 and IL-6 to promote inflammation development. In contrast, the suppressive development of Tregs and relative anti-inflammatory molecules certainly like IL-10 damaged their strong immunosuppressive function, thus both changes further promoted inflammation as well as COPD progression. Overall, we can focus on above pathways to inhibit the inflammation of COPD.

When it comes to novel and promising drug treatments for COPD with minimum side effects, we found Bufei Yishen formula and Curcumin very potential. 48 components in BYF might act on 65 targets to exert the therapeutic effect, and the therapeutic process involved IL-17 and TNF pathways. BYF inhibited the inflammation of COPD by the way of suppressing the phosphorylation of STAT3 and promoting phosphorylation of STAT5 to promote the differentiation of Tregs but inhibit the differentiation of Th17 cells. Hence, BYF decreased the levels of pro-inflammatory cytokines such as IL-1β, IL-6, TNF-α, and IL-17A secreted by Th17 cells, but increased anti-inflammatory cytokines IL-10 secreted by Tregs in the BAL fluid. As for Curcumin, it decreased the inflammation of acute lung injury not only by promoting the expression of IL-35, increasing STAT5 proportion, but also by decreasing IL-17A in lung tissue. In conclusion, both of Bufei Yishen formula and Curcumin are novel and promising drug treatments for COPD with minimum side effects, which deserve more attentions to study.

## Glossary

COPD: Chronic obstructive pulmonary disease
BYF: Bufei Yishen formula
TCM: Traditional Chinese Medicine
WHO: World Health Organization
IL-17A: Interleukin-17 A
RORγ: retinoic acid receptor-related orphan nuclear receptor γ
TIR: Toll/Il-1 Receptor
TRAF: TNF receptor associated factor
NF-κB: nuclear factor kappa-B
MAPKs: mitogen-activated protein kinases
Till: Toll/IL-11R-like loop
CBAD: C/EBP-β activating domain
Erk: extracellular signal-regulated kinase
FEV1: forced expiratory volume in 1 s
FVC: forced vital capacity
DCs: dendritic cells
EGF: epidermal growth factor
CS: cigarette smoke
ECM: extracellular matrix
HDAC: Histone Deacetylase
PAI: plasminogen activator inhibitor
HSs: healthy smokers
HCs: healthy control subjects
G-CSF: Granulocyte Colony-Stimulating Factor
NE: neutrophil elastase
TSLP: Thymic stromal lymphopoiesis
ISS: induced sputum supernatants
Ab: antibody
Ac-His H3: acetylation of histone H3
CBP: CREP-binding protein
HAT: histone acetyltransferase
FOXP: fork head/winged helix transcription factor
iNOS: inducible nitric oxide synthase
NO: inducible nitric oxide synthaseNOnitric oxide
MIP: macrophage inflammatory protein
ACP: American College of Physicians
ACCP: American College of Chest Physicians
ATS: American Thoracic Society
ERS: European Respiratory Society
LAMAs: long-acting muscarinic antagonists
LABAs: long-acting β2-agonists
STAT3: signal transducer and activator of transcription-3
PPARγ: peroxisome proliferators-activated receptor γ
NAE: NEDD8-activating enzyme
BALF: bronchoalveolar lavage fluid
